# Dissecting *Candida albicans* Infection from the Perspective of *C. albicans* Virulence and Omics Approaches on Host–Pathogen Interaction: A Review

**DOI:** 10.3390/ijms17101643

**Published:** 2016-10-18

**Authors:** Voon Kin Chin, Tze Yan Lee, Basir Rusliza, Pei Pei Chong

**Affiliations:** 1 Department of Medical Microbiology and Parasitology, University Putra Malaysia, Serdang 43400, Selangor, Malaysia; cvk717@gmail.com; 2 Department of Pathology, Faculty of Medicine and Health Sciences, University Putra Malaysia, Serdang 43400, Selangor, Malaysia; tzeyan.lee@gmail.com; 3 Department of Human Anatomy, Faculty of Medicine and Health Sciences, University Putra Malaysia, Serdang 43400, Selangor, Malaysia; rusliza@upm.edu.my; 4 Department of Biomedical Science, Faculty of Medicine and Health Sciences, University Putra Malaysia, Serdang 43400, Selangor, Malaysia

**Keywords:** *omics*, *C. albicans*, candidiasis, candida, host–pathogen interaction

## Abstract

*Candida* bloodstream infections remain the most frequent life-threatening fungal disease, with *Candida albicans* accounting for 70% to 80% of the *Candida* isolates recovered from infected patients. In nature, *Candida* species are part of the normal commensal flora in mammalian hosts. However, they can transform into pathogens once the host immune system is weakened or breached. More recently, mortality attributed to *Candida* infections has continued to increase due to both inherent and acquired drug resistance in *Candida*, the inefficacy of the available antifungal drugs, tedious diagnostic procedures, and a rising number of immunocompromised patients. Adoption of animal models, viz. minihosts, mice, and zebrafish, has brought us closer to unraveling the pathogenesis and complexity of *Candida* infection in human hosts, leading towards the discovery of biomarkers and identification of potential therapeutic agents. In addition, the advancement of *omics* technologies offers a holistic view of the *Candida*-host interaction in a non-targeted and non-biased manner. Hence, in this review, we seek to summarize past and present milestone findings on *C. albicans* virulence, adoption of animal models in the study of *C. albicans* infection, and the application of *omics* technologies in the study of *Candida*–host interaction. A profound understanding of the interaction between host defense and pathogenesis is imperative for better design of novel immunotherapeutic strategies in future.

## 1. Introduction

There are approximately 8.7 million eukaryotic species, with fungal species constituting approximately 7% (611,000 species) of the total [1]. Fungi can cause diseases ranging from cutaneous skin infections to lethal acute or chronic infections of deep tissues. There are an estimated 600 fungal species that are human pathogens, including *Candida* species [2].

*Candida* originates from the taxonomical division of ascomycetes and is a fungal genus with the potential to cause life-threatening systemic infections. It has been characterized as a white asporogenous yeast capable of forming pseudohyphae or hyphae. Currently, there are seven medically important *Candida* species, *C. albicans*, *C. tropicalis*, *C. glabrata*, *C. parapsilosis*, *C. stellatoidea*, *C. krusei* and *C. kefyr*, with *C. albicans* being the most frequent causative agent afflicting humans [3].

Since the 1970s, the incidence of *Candida* infection has increased steadily. This increment is caused by a global increase in the number of patients who are prone to infection and an improvement in clinical procedures to identify yeasts as the causative agents of nosocomial infections [4]. In immunocompromised individuals, the development of systemic candidiasis is a serious disease often associated with mortality [5].

Candidiasis is the fourth most identified cause of nosocomial bloodstream infections [6], while *Candida* species are the fourth most common cause of bloodstream infections in hospitals in the United States, with crude mortality rates of up to 50% [7,8]. Of all *Candida* species, *C. albicans* remains the most common species of bloodstream infections in hospitals. *C. albicans* is responsible for 59% of nosocomial candidemia and for 55% of bloodstream infections [9,10,11].

Not surprisingly, the incidence of candidiasis is the highest in intensive care units (ICU) among patients admitted to hospitals, and accounts for up to 15% of all nosocomial infections [12]. Bouza et al. reported that an estimated 33%–55% of all candidemia cases occur in the ICU, with mortality rates ranging from 5% to 71% [13]. Schelenz (2008) reported that *Candida albicans* is the predominant species causing candidemia in ICU patients in the United Kingdom [9]. In addition, Enoch et al. reported that *C. albicans* is responsible for 79.4% of candidaemias [14]. Candidemia in ICUs is associated with increased total hospital costs and length of stay, which imposes an extra financial burden on both individuals and the government.

Hence, the high frequency of *Candida* infection constitutes a serious clinical problem worldwide. The high mortality of *Candida*-related infections in immunocompromised patients is a perpetual problem caused by the difficulty of diagnosing and treating invasive or systemic candidiasis; thus there is a dire need for the development of sensitive and specific diagnostic methods and appropriate strategies for candidiasis treatment. It is hoped that this could be achieved through a deeper understanding of fungal virulence as well as host immune response.

## 2. Pathogenesis of *Candida albicans*

Host tissue invasion is a prerequisite for *Candida* species pathogenicity and is mediated by surface-expressed adhesion molecules, particularly specific ligand-receptor interactions and nonspecific interactions that enable *Candida* species to attach to a range of eukaryotic tissues [15]. A comparative study on the adherence of different *Candida* spp. to human epithelial cells was conducted and it was found that *C. albicans* and *C. tropicalis* adhere much more strongly than relatively less virulent strains like *C. krusei* and *C. guilliermondii* [16]. Adherence of the yeast to mucosal epithelial cells is a necessary component of the infective process with *C. albicans*. *C. albicans* has a specialized set of proteins known as adhesins that enable it to attach to a range of eukaryotic tissues [15]. The important components of adhesins are agglutinin-like sequence (ALS) proteins and hypha-associated GPI-linked protein (Hwp1). There are eight members of the ALS proteins (Als1–7 and Als9). Out of these eight members, Als3 is especially important for adhesion [17,18,19]. The *ALS3* gene expression is upregulated in oral epithelial cells in vitro and in vivo vaginal infection with *C. albicans* [20,21]. Furthermore, Als3 is demonstrated to contribute towards biofilm formation [22]. More recently, Als3 was found to be an invasin along with Ssa1 [17,23]. Ssa1, a cell-surface expressed member of the heat shock protein 70 (Hsp70) family, and *ALS3* bind to host E-cadherin, which could lead to endocytosis by a clathrin-dependent mechanism [17,24]. Meanwhile, Hwp1 serves as a substrate for mammalian transglutaminases and formed covalent bonds with human buccal epithelial cells that covalently link *C. albicans* hyphae to host cells. Previous studies with an Hwp1-deficient mutant and an Hwp1 knockout mutant of *C. albicans* displayed reduced adherence to buccal epithelial cells and reduced mortality in an intravenous challenge murine model [25,26,27].

*C. albicans* can exist as yeasts or elongated filaments (true hyphal or pseudohyphal) depending on environmental conditions, nutrients, and temperature [28,29]. This enables *C. albicans* to become a successful pathogen that can infect a variety of tissues in a mammalian host. Both yeast and filamentous forms of *C. albicans* can be observed in infected tissues during infection. Studies showed that mutants of *C. albicans* that lack either the yeast or filamentous form have reduced virulence in animals. The yeast form of *C. albicans* is involved in dissemination within tissues and to other hosts and its form is maintained through the repression of the filamentous form by several transcriptional factors such as *Tup1*, *Nrg1*, and *Rfg1* [30,31]. Nonfilamentous *C. albicans* strains with defective transcriptional factors, such as *efg1* and *cph1*, have been demonstrated to be less virulent in mouse infection models [32]. Filaments of *C. albicans* are required for tissue damage and escape killing by macrophages. Carlisle et al. and Lo et al. found that tissue invasion is associated with *C. albicans* filamentous forms and mutants of *C. albicans* compromised in filamentous form have reduced virulence [32,33]. Similarly, the homozygous null mutant of *Tup1* compromised in filamentous expression was unable to colonize organs in an experimental disseminated mouse model [34,35], which further suggested that the filamentous form of *C. albicans* is critical in tissue colonization and invasion. Furthermore, hyphal cells have stronger adherence capacity due to the expression of ALS adhesins and display greater invasiveness into tissues. In addition, hypha-associated proteins such as *Ece1*, *HGC1*, and *Hyr1* are important virulence factors. For example, the ablation of *HGC1* in cells that grow normally in yeast form leads to failure in the hyphae production. This is because this protein encodes a hypha-specific G1 cyclin-related protein [36]. Moreover, the yeast and hyphal forms of *C. albicans* provoke different immunological responses. The yeast form favors the formation of T-helper type 1 (Th1) cells, which have protective effects, while filamentous forms favor the formation of T-helper Type 2 (Th2) cells, which have been linked to disease progression [37].

Phenotypic switching allows for a transition between alternative cell states and is crucial for pathogenic fungi to colonize and infect different host niches. *Candida albicans* displays a white-opaque switch important in regulating sexual mating [38,39], metabolic preferences [40], and interaction with the mammalian host immune system [41,42]. In the two alternative phenotypic states, white cells are round, smooth, and domed colonies, whereas opaque cells are elongated, bean-shaped, flatter, and darker colonies [43]. The switching between white and opaque states is regulated by the *WOR1* gene. White cells are unable to form opaque colonies in the absence of *WOR1*, whereas overexpression of *WOR1* forces cells to switch to the opaque state [44,45,46]. Transcriptional studies reveal that when white and opaque states are compared, there are more than 450 genes that are differentially regulated [40,47]. Moreover, the differences in these transcriptional profiles between the white and opaque form affect *C. albicans* mating efficiency, pathogenicity, and biofilm formation [48]. For example, white cells have higher virulence in an intravenous challenge mouse model, while opaque cells appear to be more efficient at the colonization level in a cutaneous model of infection [49,50]. Furthermore, white–opaque switching may be an adaptive mechanism to assist *C. albicans* cells in evading the host immune system as white and opaque cells are differentially phagocytosed by macrophages [42]. On the other hand, the morphological switching between the yeast and hyphal form contributes to mature biofilm formation, which is the lethal outcome in *Candida* infection [51].

Right after adhesion of *C. albicans* to the cell surface, *C. albicans* hyphae secrete hydrolases to facilitate active penetration into host cells and enhance the efficiency of extracellular nutrient acquisition [52,53]. Fungi such as *C. albicans* produce secreted aspartyl proteinases (SAPs), which play a role in supplying nutrients for the *Candida* cells through protein degradation, facilitating penetration and invasion into host tissues in addition to evading immune responses [54,55]. This class of enzyme has been demonstrated to be involved in candidiasis [56]. Catalytic properties of SAPs lie in the conserved sequence of Asp-Thr-Gly as this sequence provides one-half of the catalytic machinery of these enzymes [57]. The enzyme of this class is also defined from the Thr-Gly sequence following the Asp, which is essential for the formation of a unique catalytic conformation. Interestingly, *C. albicans* had been reported to possess a family of 10 genes encoding SAPs, which might explain the pathogenesis of the disease [58]. Correspondingly, SAPs have also been utilized in the diagnosis of systemic candidiasis as markers of invasive candidiasis via competitive binding inhibition enzyme-linked immunosorbent assay (ELISA) [10]. The key advantage of using SAP markers of candidiasis lies in their ability to differentiate simple colonization from invasive disease. In light of SAP as a virulent factor, Vilanova et al. exploited *C. albicans* SAP2 as a potential vaccine candidate in preventing systemic candidiasis in BALB/c mice [59]. These authors noted that the use of SAP2 protein conjugated with alum adjuvant conferred efficient immune protection by a 20-fold decrease in the colonization of kidneys. In addition, the protection also correlated with an increase in serum antibodies, particularly immunoglobulin G towards SAP2. Based on existing literature on SAP-deficient mutants, SAP1, SAP2, and SAP3 have been shown to contribute significantly to tissue damage and invasion of oral epithelium, while SAP4, SAP5, and SAP6 are important for systemic infections caused by *C. albicans* [60].

Another family of *Candida* virulence enzymes are the phospholipases, which consist of four different classes (PLB-A, PLB-B, PLB-C, and PLB-D) [61]. However, only five members of class B (*PLB1-5*) are extracellular and may contribute to pathogenicity. Phospholipase B expression has been detected immunologically in gastrointestinal and systemic infection and in mucosal models [58]. The mechanisms of action of phospholipase enzyme are disrupting and breaking down the phospholipids in host cell membranes, which in turn aid adherence and penetration and finally invasion of host cells [62]. The family of lipases consists of at least 10 members (*LIP1-10*) [63,64]. *LIP5*, *LIP6*, *LIP8*, and *LIP9* expression was detected in a mouse model of *C. albicans* peritonitis [64]. A study reported that *C. albicans* strains with lack of *Lip8* expression displayed reduced virulence in infected mice, suggesting that lipases are involved in altering *C. albicans* pathogenicity [65].

Biofilms of *C. albicans* are multicellular communities of yeast, pseudohyphae, and hyphae surrounded by extracellular matrix, which form upon adherence to biotic or abiotic surfaces. Biofilms are formed in a sequential process. These include the adherence of yeast cells to the substrate, followed by proliferation of these yeast cells and hyphal cells in the upper part of the biofilm, which is then followed by accumulation of extracellular matrix material and dispersion of yeast cells from the biofilm complex [66,67]. There are several genes that induce or regulate the formation of biofilm. These transcription factors include Bcr1, Tec1, Efg1, and Hsp1 [68]. In addition to that, Nobile and co-workers reported on novel transcriptional factors involved in regulating biofilm formation, which include Rob1, Ndt80, and Brg1 [69]. Furthermore, defective biofilm formation resulting from the deletion of any of these transcriptional regulators (ROB1, BCR1, NDT80, EFG1, TEC1, or BCR1) was observed in an in vivo rat infection model [69].

Biofilm formation of *C. albicans* is a huge problem in clinical settings as biofilms on implanted devices such as catheters, pacemakers, and prosthetic devices enable the fungal cells to have direct access into the bloodstream for dissemination and establishing life-threatening systemic infection. The dispersion of yeast cells from the mature biofilm has been demonstrated to directly contribute to virulence in an experimental mouse model of systemic infection [67]. Another concern about biofilm formation in clinical settings is that they are highly resistant to antifungal drugs and host immune factors in comparison with planktonic cells. Factors such as upregulation of efflux pumps, upregulation of oxidative stress, changes in membrane sterol composition, and increased cell density might contribute to the resistance of biofilm to antifungal drug treatment [68].

Contact sensing and thigmotropism (response to both abiotic and biotic surfaces) are important for pathogenicity. The environmental factors provide signals to trigger the hypha and biofilm formation in *C. albicans*. The yeast cells will then switch to hyphal growth upon surface contact [70]. These hyphae have the potential to invade into the substratum when in contact with substrates such as mucosal surfaces or agar. Biofilm formation can also be induced due to contact with solid surfaces [70].

pH sensing has also been discovered to be vital in pathogenicity mechanism. *C. albicans* must get used to pH changes in its surroundings as it is exposed to a range of pH from alkaline to acidic in the human host [71]. The two cell wall β-glycosidases, namely *PHR1* and *PHR2*, are important proteins in the adaptation to pH changes. *PHR1* and *PHR2* are expressed in neutral-alkaline pH and acidic pH, respectively [72,73].

Clearly, *C. albicans* has evolved into a highly successful microorganism with the ability to colonize and infect the human host through a plethora of virulence determinants and adaptation mechanisms. Hence, a better understanding on the pathogenicity of *C. albicans* during the infection process will enable us to spawn new ideas on improving the diagnosis of fungal infection or developing new antifungal regimens. For example, *Candida* virulence factors that are crucial in dimorphism, adhesion, and tissue invasion as well as hydrolytic proteases have been proposed as attractive targets and promising antifungal therapy [74,75].

## 3. *C. albicans* Infection in Animal Models

Several animal hosts have been used to investigate events such as invasion and colonization by *Candida* species. These common host systems include mice, fruit flies, nematodes, and zebrafish. Each model has its own advantages and limitations. Hence, it is important for researchers to choose a suitable animal model that fits the experimental design and hypothesis that are being tested. In this review, we will discuss some of the animal models involved in the study of *C. albicans* infection.

Several mini-host models, viz. the fruit fly (*Drosophila melanogaster*), nematode (*Caenorhabditis* *elegans*), and wax moth (*Galleria mellonella*) [76,77,78], were adopted for *C. albicans* study. The *D. melanogaster* model has been employed in many studies to study, for instance, *Candida* virulence and mutants and the efficacy of antifungal drugs [77], to investigate cell-mediated innate immunity against *C. albicans* [79], and to probe the links between nutrient availability in infected hosts and *Candida* virulence [80]. These studies suggest that *D. melanogaster* is a suitable model for large-scale studies of antifungal drug activity, virulence mechanisms, and cell-mediated innate immunity in candidiasis. On the other hand, the *C. elegans* model has also often been used in the screening and development of new antifungal chemical compounds [81], to study *Candida* virulence factors [68], and to examine antifungal innate immunity [82]. Like the *D. melanogaster* and *C. elegans* models, the *G. mellonella* model has been employed in many studies, including experiments on *Candida albicans* mutants and virulence [83,84], *Candida* virulence factors [85,86,87], *C. albicans* virulence transcriptional factors [88], photodynamic therapy (PDT) as a treatment regimen for *Candida* infections [89], and the evaluation of the efficacy of antifungal agents [90]. Overall, the adoption of these mini-hosts in *C. albicans* infection studies provides a number of advantages. Firstly, these mini-hosts are relatively inexpensive care systems in contrast to animal models. Secondly, they are highly amenable to large-scale studies of virulence factors with well-developed molecular tools. In addition, they are an efficient high-throughput drug screening platform and have conserved innate immunity. However, the major drawback of these mini-hosts is that they lack adaptive immunity, and so cannot reflect the real situation of complex fungal pathogenesis and host–pathogen interaction.

Besides these mini-hosts, animal models are used to study the molecular and cellular basis of pathogenesis of candidiasis and allow the ideal study of host–pathogen interactions under a tightly controlled environment as the tissue samples are easily harvested unlike studies on human patients, which present a myriad of complexities. Moreover, the patterns of tissue damage in targeted organs and overall disease pathology and progression in animal models are closely related to humans’ specific immune and hormonal responses [91,92].

The murine model is one of the predominant animal models in *C. albicans* infection studies due to its direct implications for human systems [93]. Murine models have been widely used in the study of oral, vaginal, and systemic *Candida* infection as they mimic human *Candida* infection [94,95,96]. The murine challenge intravenous model is considered the “Gold Standard” in the study of systemic *Candida* infection [97]. The process for establishing this disseminated infection involves the intravenous injection of *Candida* cells into the lateral tail vein of mice, which mimics *Candida* bloodstream infection in humans. This model can be used to verify genetic determinants of *Candida* virulence, host–pathogen interaction, and the efficacy of new antifungal drugs. The murine model has also been widely used in the study of oral candidiasis. The advantage of the murine oral candidiasis model is that it only shows typical lesions that are related to local symptoms of oral candidiasis, allowing more precise quantification of the disease burden [94]. In addition, employment of a murine model further enhances our understanding of host–pathogen interactions in oral candidiasis. For example, Th17 helper T cells and their secreted cytokine IL-17 have been revealed to have a protective role against oral candidiasis. The benefits of murine models of candidiasis include the high similarity to human infection due to the immune system response, which is far superior than using mini-hosts; and the ability to study whole-organ and systemic host response as well as disease pathology. However, some of the drawbacks of adopting mice models include excessive experimental costs, inherently unethical practices, and a limited number of offspring produced for large-scale studies.

Other than mini-hosts and murine models, zebrafish (*Danio rerio*) have also been used in the study of *Candida* pathogenesis and virulence. Embryonic zebrafish were used to study *C. albicans* morphology changes and its link to gene expression and early host response towards this pathogen [98]. Since a zebrafish embryo is transparent, researchers can perform real-time visualization of host–pathogen interactions [99], thus enabling researchers to monitor and track the changes simultaneously in real time in both the pathogen and host, which in turn generates a more precise and accurate interpretation of the host–pathogen interactions. Meanwhile, Brothers et al. used larval zebrafish to study the role of phagocyte oxidase in limiting filamentous growth of *C. albicans* and reported that NADPH oxidase is essential for the control of *C. albicans* filamentation in vivo [100]. Chen et al. also used zebrafish to study host–pathogen interactions in *C. albicans* [101]. In a subsequent study, Chen et al. utilized zebrafish eggs to study the gene functions in *C*. *albicans* and proposed that the zebrafish model is cost-effective and time-saving [102]. The advantage of using the zebrafish model is that it is less invasive compared to animal models. Since zebrafish larvae and embryos are transparent in nature, non-invasive imaging techniques can be used for studies like genetic manipulation or pharmacological treatment. These less intrusive techniques can minimize animal suffering and improve experimental outcomes. Besides that, the zebrafish has a high reproductive rate, which makes it suitable for large-scale studies, and the availability of comprehensive molecular tools for analyzing the results produced makes it more economical due to low maintenance costs [103]. Moreover, unlike mini-hosts that lack adaptive immunity, zebrafish comprises both innate and adaptive immunity, and mimics mammals in the context of anatomy, physiology, and genetics [104].

Therefore, it is imperative to understand the intricate interaction between host and pathogen through the adoption of animal models infected with *Candida* species as it will enable us to consolidate our knowledge of how the pathogen interacts with and influences host cell function, which simulates the transition from asymptomatic colonization to symptomatic infection in humans. This will eventually lead to the development of effective antifungal drugs for treating *Candida* infection.

## 4. *Omics* Perspective on *Candida*–Host Infection

Due to advancements in the areas of innate and regulatory immunology, host–pathogen interaction has become the “epitome” of biomedical research. In addition, with the advancement of *omics* technology and the development of advanced systems biology tools, researchers can approach host–pathogen interaction during *Candida* infection in an unprejudiced and quantitative manner.

The host–pathogen interaction is a very complicated process, and much remains to be fully elucidated to dissect the different aspects of *C. albicans* virulence and unravel its interactions with the innate immune system and the contribution of each of these events to *C. albicans* pathogenesis, which may ultimately lead to better therapeutics that can target the cellular response to infection.

## 5. Transcriptomics

There have been numerous transcriptomic studies on the interactions between host and *C. albicans.* By using microarray analysis, Kim et al. reported that the co-culture of human monocytes with *C. albicans* resulted in upregulation of genes involved in production of proinflammatory cytokines, interleukins, and tumor necrosis factor-α [105]. In addition, primary human endothelial cells were found to upregulate 56 genes that predominantly consist of chemotaxis, cell death, and cell signaling related genes against *C. albicans* in vitro [106]. Interestingly, a study by our group comparing the transcriptomic profiles of low- and high-density *C. albicans*-infected human umbilical vein endothelial cells (HUVECs) revealed that genes involved in apoptosis and cell death were significantly differentially expressed. Our results challenged the conventional belief that the yeast form is avirulent and merely plays a role in dissemination [107]. Using zebrafish, Chen et al. studied the host–pathogen interaction in *C. albicans* and categorized *C. albicans* infection into three stages, namely adhesion, invasion, and damage. In addition, there is activation of the filamentous formation during invasion phase and iron scavenging roles during the damage phases in *C. albicans*. The expression of the majority of the immune-related genes was seen during the progression of infection from invasion to damage [101].

On the other hand, in whole-organ response during systemic *C. albicans* infection, MacCallum (2009) demonstrated that the host response in the kidney against both virulent and attenuated *C. albicans* strains mainly involved acute-phase response, coagulation cascade, and complement. In addition, MacCallum (2009) reported that infection with a virulent strain of *C. albicans* induces host immune responses in the kidney, mainly innate immunity, while infection with an attenuated strain of *C. albicans* was associated with non-immune responses such as metabolic pathways. MacCallum et al. (2009) deduced that early-expressed chemokines were important in predicting the kidney’s immunopathology during systemic *C. albicans* infection [108]. In another study by Chin et al. (2014), our research group suggested that pathogenesis of systemic *C. albicans* infection involved multiple stages and demonstrated organ-specific host immune responses, mainly innate immune response towards *C. albicans* infection [93].

Barker et al. (2008) demonstrated that infection with *C. albicans* induces specific endothelial cell responses, not confined to upregulation of host immune genes [109]. Other non-immune responses such as angiogenesis, stress response, and inhibiting apoptosis were reported, likely caused by *C. albicans*-induced damage to the endothelial cells. Mice infected with *C. albicans* were reported to have endothelial cell proliferation in the regions adjacent to microabscesses in the kidney and brain [110], and the proliferation of endothelial cells was likely caused by expression of genes that inhibit apoptosis and stimulate angiogenesis. In addition, Jose et al. (2013) reported an increase in anti-apoptotic signal, which is Bcl12 family member Myeloid cell leukemia 1 (Mcl1) [111]. Mcl1 is involved in promotion of cell viability during phenotypic transition which includes stimulation of differentiation or proliferation [112]. Another anti-apoptotic signal includes the increase in phosphorylation of nucleophosmin (NPM), a nucleolar phosphoprotein that binds the tumor suppressors p19Arf and p53. NPM is important for cell proliferation, ribogenesis and survival after DNA damage [113].

Taken together, the majority of the previous studies have shown that the innate immunity mediated by neutrophils, macrophages, and monocytes is crucial in pathogen detection, antimicrobial defenses, the activation of the adaptive immune system, and eventually controlling the disease outcome. Nonetheless, findings from Barker et al. (2008) suggested that the role of angiogenesis should be investigated in the pathogenesis of disseminated candidiasis, and the contribution of other non-immune response pathways besides classical immune response pathways should be explored further to better understand the host defense against *Candida* [109]. On the other hand, the inhibition of apoptosis could be a host immune mechanism reinforcing defenses by maintaining active and viable macrophages against *C. albicans*. However, the inhibition of apoptosis could become another *C. albicans* putative virulence factor if the infected macrophages favor the replication and dissemination of the *C. albicans* that remain alive inside them. Subsequently, it will be interesting to determine whether the anti-apoptotic response is regulated by the pathogen or by the macrophages.

By using RNA sequencing and siRNA technology, Liu et al. (2015) further improved our understanding of the interaction between *C. albicans* infection and host response. In their study, transcriptional profiles of both C*. albicans* and host cells during in vitro infection of oral epithelial and vascular endothelial cells were analyzed. A number of signaling proteins that were not previously linked with the host response to any pathogenic fungus were uncovered via a network analysis of the dataset. The authors also deduced that neural precursor-cell-expressed developmentally downregulated protein 9 (NEDD9) and platelet-derived growth factor BB (PDGF BB) govern the host–pathogen interaction by regulating the uptake of *C. albicans* by host cells [114].

## 6. Genomics

Genetic susceptibility to *Candida* diseases has generated much interest lately [115]. Smeekens and his research group have reported that *Candida* susceptibility caused by various genetic variants are found in downstream signaling molecules and pathogen recognition or cytokine receptors [116]. In addition, a genome-wide association study (GWAS) with 217 human candidemia patients performed by Kumar et al. discovered that SNPs in CD58, LCE4A, and TAGAP will elevate the risk for candidemia [117].

## 7. Proteomics

Transcriptomic studies can provide new insights on host–pathogen interaction; however, the findings from transcriptomic studies only pertain to the gene level, while certain effectors might not be expressed in cells and thus cannot fully reflect the active effectors or molecules in an organism. Therefore, proteomic analyses of the interaction of *C. albicans* with host immunity is crucial to elucidate the molecular interplay of host immune defence mechanisms, to understand how *C. albicans* can escape from host immunity, and to identify antigenic proteins during *Candida*–host interaction that could serve as diagnostic or prognostic markers and therapeutic interventions in the near future.

Vialás et al. first established a database named Proteopathogen, a protein database that focuses on *C. albicans*–host interaction [118]. Proteopathogen initially emphasized *C. albicans* and its interaction with macrophages and a subsequent version, Proteopathogen2, expanded on that concept and included the adoption of the HUPO-PSI standards [119]. It also includes information on proteomics experimental data and proteomic workflows. Martínez-Solano et al. studied the differential expression of proteins in macrophages upon interaction with wild-type strain *C. albicans* SC5314, in a murine macrophage cell line RAW 264.7 [120]. In the study, the authors reported that the expression of LyGDI, annexin I, Hspa5, l-plastin, and tropomyosin 5 were augmented while the expression of eif3s5, grp58, Hspa9a, Hsp 60, and Hspa8a were reduced. These differential findings indicate that many processes are affected, viz. protein biosynthesis and refolding, oxidative responses, and cytoskeletal organization. In another study by Martínez-Solano et al., the authors studied the interaction of macrophage RAW 264.7 with heat-inactivated *C. albicans* cells and revealed that the overall response involved was anti-inflammatory. The authors also postulated that the differential proteomic response from a macrophage could depend on the status of *C. albicans* cells, either alive or heat-inactivated [121]. Reales-Calderón et al. investigated the interaction between RAW 264.7 macrophages and *C. albicans* through quantitative proteomics and phosphoproteomics approaches. Reales-Calderón and co-workers reported that macrophage responses against *C. albicans* involved the inflammatory proteins and expression of proteins related to oxidative stress including receptors, cytoskeletal proteins, mitochondrial ribosomal proteins, and transcription factor activators. Furthermore, the authors also identified proteins related to apoptosis in the study and through analysis of apoptotic markers; anti-apoptotic signals were strong and hence the authors suggested that, apart from inflammation, the apoptosis pathway is crucial in host defense against *C. albicans* [122]. In the following year, Reales-Calderón and co-workers [123] studied the differential proteome expression between human M1 and M2 macrophages upon stimulation with *C. albicans.* The findings revealed that the substantial differences between M1 and M2 macrophages were found in metabolic and cytoskeletal rearrangement routes. In addition, the authors found that fructose-1,6-biphosphatase 1, an important enzyme involved in gluconeogenesis, is upregulated in M1 macrophages. Furthermore, upon interaction with *C. albicans*, the authors observed an M1-to-M2 switch in polarization. This switching exacerbates *Candida* pathogenicity by diminishing the immune responses, which in turn increase fungal survival and colonization or an attempt by the host to mitigate the damage caused by inflammation [123].

Kitahara et al. analyzed the escaping behavior of *C. albicans* from macrophages by using mixed and quantitative proteome analysis in isolation [124]. The authors identified five proteins from macrophages and 237 proteins from *C. albicans* as candidate interaction-specific molecules. The authors reported that macrophage-induced *C. albicans* proteins were linked with stress response, membrane synthesis, glucose generation, and other unknown functions. Meanwhile, *C. albicans*-induced macrophage proteins were linked with a chaperone and apoptosis. The authors postulated that *C. albicans* can endure the harsh environment inside macrophages through upregulation of stress-related proteins, while the escape of *C. albicans* from macrophages could be associated with the production of glucose from a β-oxidation pathway and downregulation of chaperone HSPA1A- and apoptosis-associated protein, NOA1-syntheses, in macrophages [124]. In summary, proteome research on host–pathogen interaction with respect to *Candida* infection is still an emerging field. More comprehensive and large-scale research is needed in the future to unravel the mechanisms of pathogenicity and *Candida* virulence.

## 8. Computational Systems Biology

The advancement of *omics* technologies in previous years augments the demands of computational assistance to analyze *omics* data. In fungal immunity, these *omics* data may include transcriptomic, proteomic, and metabolomic findings to generate interaction networks at the host–pathogen level, which can be used to predict the outcome of infection. Furthermore, datasets from host–pathogen interaction were obtained from direct experimentation quantification and there was no linkage between these datasets. Hence, computational systems biology provides a platform to link these datasets from various parameters to identify key characteristics of host–pathogen interactions.

Computational systems biology is an emerging area in fungal immunity study. Hünniger and co-workers (2014) investigated the innate effector mechanisms and *Candida albicans* immune escape in human blood through experimental and computational models. Firstly, the authors analyzed the innate immune response against *C. albicans* by adding fungal cells into human whole blood and found that the predominant immune response was associated with neutrophils, with a minority mediated by monocytes. The authors then developed a virtual *C. albicans* infection model that permitted detailed and quantitative predictions on the dynamics of *Candida*–host interaction in whole blood. The time-resolved data were designed and simulated by a state-based modeling approach that was combined with the Monte Carlo method of simulated annealing to identify the main components of antifungal immunity and to obtain quantitative predictions on a priori unknown transition rates. The authors concluded that in human blood, neutrophils were predominant in killing and eliciting an immune response against *C. abicans*, mediated through intracellular killing and phagocytosis; and that the escape of *C. albicans* from immune response was independent of filamentation, inactivation, or exhaustion of innate immune cells. The resistance of *C. albicans* cells to phagocytosis could lead to dissemination in a bloodstream infection. In addition, the authors also suggested that this experiment–model–experiment cycle is useful in quantitative analyses of host–pathogen interaction in a complex environment like human blood [125].

On the other hand, Dühring et al. [126] gave an overview of host defense strategies, which include immunological mechanisms and general stressors as well as fungal evasion mechanisms, and the adoption of computational systems biology approaches to investigate these complex interactions through agent-based models and game-theoretical methods. In addition, Durmuş et al. summarized the available literature on the computational analysis of PHI (Pathogen–Host Interaction) networks, including PHI network inference using omics data, computational prediction of PHIs, text mining of PHI data and mathematical modeling, and bioinformatic analysis of PHIs [127]. Remmele and his co-workers constructed a PHIs network analysis of *C. albicans* and reported that Eno1, phospholipase B (PLB1), Hsp70, and pyruvate kinase (CDC19) are among the *Candida* virulence factors that interact with host factors CD4, Alb and amyloid beta (A4) precursor protein (APP), Toll-like receptor 2 (Tlr2), and epidermal growth factor receptor (Egfr), respectively. This integrated network will be useful for high-throughput analysis of host–pathogen transcriptome and proteome data in the future [128]. On the other hand, Dix et al. constructed a random forest classifier to determine whether a sample contains a fungal, bacterial, or mock infection by using transcriptomic data obtained from the interaction of bacteria pathogens *Staphylococcus aureus* and *Escherichia coli* and the fungal pathogens *Candida albicans* and *Aspergillus fumigatus* in a human whole-blood model [129]. This analysis provides new insights into systemic host responses and may ultimately lead to the development of new anti-microbial regimens. Lehnert et al. [130] constructed a bottom-up modeling approach, from a state-based model to an agent-based model, to simulate *C. albicans* infection in a human whole-blood model. The authors reported that polymorphonuclear neutrophils (PMN) cells are important effector cells in killing *C. albicans* in human blood. The authors also surmised that a systemic medicine approach that utilizes the predictive power of virtual infection models will play a crucial role in infectious disease diagnosis in the future [130].

The potential of computational tools in predicting the host survival in *C. albicans* infection was explored by Peltz and his research group through a combination of computation and experimental models. In the study, a next-generation computational genetic mapping program with advanced features was developed. The computational analysis revealed that early classical complement pathway components (C1q, C1r, and C1s) could affect the survival of mice in a murine genetic model of hematogenous *C. albicans* infection. Experimental study further substantiates the computational results, wherein the binding of serum C1 to *C. albicans* and the survival of chromosome substitution strains was greatly influenced by C1rs alleles. The authors concluded that a combination of a next-generation computational genetic mapping program and an experimental murine genetic model accurately predicted survival after *C. albicans* infection, which involves an interaction between C5 and C1r/s alleles. Furthermore, it was also suggested that this combinatorial, conditional genetic model could enhance our knowledge of the genetic factors affecting susceptibility to neurodegenerative, autoimmune, and infectious diseases [131].

In summary, computational modeling of PHIs networks is still in its infancy due to a scarcity of data. However, the modeling of large amounts of data obtained from transcriptomic, proteomic, and metabolomic studies can help us to better understand the mechanism of infection, discover biomarkers for disease diagnosis, and identify novel therapeutic agents for treating infectious diseases.

## 9. Challenges Apart from Omics and Systems Biology Approaches

Owing to the advancement of *omics* technology and systems biology, there are still challenges in the analysis of the host–pathogen system. Krüger et al. [132] addressed some of the challenges that need to be considered when conducting proteomic analysis of the direct interaction between phagocytic immune effector cells and fungi, including the enormous complexity of proteins that include expanded dynamic ranges, selection of suitable blood donors for the study, selection of an appropriate multiplicity of infection (MOI) to avoid downgrading of the result of relative quantitative analysis, the demands on isolation procedures for immune effector cells, the demands on culture conditions for immune effector cells, and the homogeneity of the cell suspensions used for inoculation [132].

Transcriptomic profiles might be useful in fungal diagnostics in the future. Several studies reported that *Candida* infection can be discerned from bacterial infection depending on the induced transcriptome profile [133,134]. However, certain challenges need to be taken into consideration. The transcriptomic profiles should be replicated in independent studies and involve large samples of candidemia patients for validation. Furthermore, an additional challenge is that transcriptome studies are quite time-consuming and costly. The data generated from transcriptome studies is complicated, and standardization and validation are required between each study [135].

Careful design of experimental protocols is needed when fungal studies are involved. This is due to the unique fungal anatomy, especially the fungal cell wall, which may result in the inefficacy of some experiments. The fungal cell wall may interfere with fungal cell lysis, which could lead to incomplete lysis of fungal cells. Moreover, Culibrk et al. (2006) found that the fungal cell wall may interfere with the metabolic labeling necessary for techniques such as SUnSET or SILAC [136]. On the other hand, *omics* and systems biology studies involve the usage of new and high-throughput technologies. Many of these techniques have been recently reported and are yet to be validated for accuracy and specificity. Hence, challenges arise when these new technologies are employed in the laboratory.

## 10. Conclusions

*C. albicans* remains one of the most common fungi, causing high mortality and morbidity in immunocompromised patients. This review article highlights the important factors contributing to *C. albicans* virulence, the advantages and drawbacks of various animal models adopted for *C. albicans* pathogenesis, and the application of *omics* technologies, particularly transcriptomics, proteomics, and computational systems biology in *Candida*–host interaction. As systems biology encompasses all *omics* approaches, it is highly valuable for unraveling the complexity of *Candida*–host interaction and for the design of improved therapeutic regimens. As we look to the future, we can anticipate an exponential increase of data from the application of these new technologies, ultimately aiding us in attaining a more holistic understanding of the complex relationship between fungi and humans.
